# Relationship of clinical symptoms with biomarkers of inflammation in pediatric inflammatory bowel disease

**DOI:** 10.1007/s00431-016-2762-2

**Published:** 2016-08-29

**Authors:** Daniël R. Hoekman, Kay Diederen, Bart G. P. Koot, Merit M. Tabbers, Angelika Kindermann, Marc A. Benninga

**Affiliations:** Department of Pediatric Gastroenterology and Nutrition, Academic Medical Center, Meibergdreef 9, 1105 AZ Amsterdam, The Netherlands

**Keywords:** Crohn’s disease, Ulcerative colitis, Inflammatory bowel disease, Disease activity, Fecal calprotectin

## Abstract

In adult inflammatory bowel disease (IBD) patients, there is a strong discrepancy between symptoms and biomarkers of inflammation. Data on pediatric IBD patients are conflicting. Therefore, we aimed to investigate the relationship between clinical symptoms and biomarkers of inflammation in pediatric IBD. Patients aged <18 years with previously diagnosed Crohn’s disease (CD) or ulcerative colitis (UC) were included. Clinical disease activity was determined using the abbreviated Pediatric CD Activity Index (aPCDAI) or Pediatric UC Activity Index (PUCAI). Biochemical disease activity was assessed using fecal calprotectin (FC) and C-reactive protein (CRP). In total, 127 patients (62 male; median age 14.9 years) were included (82 CD, 45 UC). FC correlated weakly with total aPCDAI score (*r*_s_ = 0.32; 95 % CI 0.12–0.51; *p* = 0.003) and total PUCAI score (*r*_s_ = 0.36; 95 % CI 0.07–0.62; *p* = 0.015). Only aPCDAI components abdominal examination and perirectal disease and PUCAI component activity level had a significant correlation with levels of FC. CRP correlated weakly with total aPCDAI score (*r*_s_ = 0.28; 95 % CI 0.05–0.46; *p* = 0.012) and aPCDAI components abdominal examination and activity level. No significant correlation was observed between CRP and total PUCAI score (*r*_s_ = 0.01; 95 % CI −0.34–0.29; *p* = 0.961) or individual PUCAI components.

*Conclusion*: There is a strong discrepancy between clinical symptoms and biomarkers of inflammation in children with IBD.

**Table:** 

**What is Known:** • *A substantial proportion of asymptomatic pediatric inflammatory bowel disease (IBD) patients have elevated biomarkers of inflammation*. • *There is a strong discrepancy between symptoms and biomarkers of inflammation in adults with IBD*.
**What is New:** • *Clinical symptoms are only weakly associated with levels of fecal calprotectin and serum C-reactive protein in children and adolescents with previously diagnosed IBD*. • *Similarly to adult IBD patients, there is a strong discrepancy between clinical symptoms and biomarkers of inflammation in children with IBD*.

**Electronic supplementary material:**

The online version of this article (doi:10.1007/s00431-016-2762-2) contains supplementary material, which is available to authorized users.

## Introduction

Traditionally, treatment of inflammatory bowel disease (IBD) was mainly guided by symptoms such as abdominal pain, bowel habits, and general well-being. Symptomatic treatment, however, may not improve long-term outcome or slow disease progression [27], possibly because symptoms may not accurately reflect the underlying inflammatory process [19]. Therefore, mucosal healing (often defined as the complete resolution of macroscopic inflammation on endoscopy) is increasingly advocated as a therapeutic target in IBD [27]. Indeed, mucosal healing is a predictor of long-term outcome of both Crohn’s disease (CD) and ulcerative colitis (UC) [31, 32]. However, due to its invasiveness and costs, endoscopy is not ideal for frequent monitoring of disease activity. Therefore, in current practice, biomarkers such as fecal calprotectin (FC) and serum C-reactive protein (CRP) are frequently used as surrogate markers of endoscopic IBD activity. Especially, levels of FC have a strong correlation with endoscopic disease activity [26, 7, 6, 36]. It can, however, be argued that complete normalization of FC—potentially indicating complete resolution of macroscopic and microscopic inflammation—is a therapeutic target beyond mucosal healing. In support of this, recent studies in adults have shown that even in the absence of endoscopic signs of disease activity, levels of FC are predictive of long-term outcome [28, 20]. Furthermore, a therapeutic algorithm based on levels of FC alone did improve medium-term outcome in adults with IBD [24, 26]. However, the optimal target of treating IBD remains to be established, and a gold standard for monitoring IBD is currently not available [26].

A substantial proportion of asymptomatic pediatric IBD patients have elevated biomarkers of inflammation [35]. Data on adult IBD patients indeed indicate that there is a strong discrepancy between clinical symptoms and biochemical markers of inflammation [3, 36]. Data on pediatric IBD are conflicting, with reported correlations between symptoms and FC levels ranging from absent (*r* = 0.0) [12, 33] to strong (*r* = 0.7) [13] in CD and from weak (*r* = 0.3) [15] to strong (*r* = 0.8) [2] in UC.

We aimed to investigate the relationship of clinical symptoms with biochemical markers of inflammation in a random sample of children and adolescents who were previously diagnosed with and treated for IBD.

## Methods

### Patients

In this cross-sectional, observational study, we aimed to include children and adolescents (aged <18 years) with an established diagnosis of CD or UC according to revised Porto criteria [17], who visited the outpatient pediatric gastroenterology clinic of the Emma Children’s Hospital/Academic Medical Center, Amsterdam, the Netherlands. Patients with IBDU, an ileo- or colostomy, or a colectomy with ileal pouch-anal anastomosis were excluded. Approval from the local Medical Ethics Review Committee was obtained.

### Data collection

Patient characteristics were obtained from patients’ medical records, including age, sex, age at diagnosis, disease duration, disease location and behavior, the presence of (previous) growth impairment, current medication, and previous IBD-related surgery. Paris classification was used to classify IBD phenotype [16].

Clinical disease activity was determined using the Pediatric Ulcerative Colitis Activity Index (PUCAI) for UC patients [37] or the abbreviated Pediatric Crohn’s Disease Activity Index (aPCDAI) for CD patients (Online Resource) [18, 34]. The PUCAI consists of six clinical items, with scores ranging from 0 to 85, with cutoff scores for remission (<10 points), mild (10–34), moderate (35–64), and severe disease (≥65) [37]. The aPCDAI consists of six clinical items, with scores ranging from 0 to 70, with cutoff scores for remission (<10 points), mild (10–25), moderate (26–39), and severe disease (≥40) [34, 18].

Biochemical disease activity was assessed using FC and serum CRP levels at the time of assessing clinical disease activity. Fecal calprotectin concentrations were measured using an enzyme-linked immunosorbent assay (Bühlmann Laboratories, Switzerland). The respective lower and upper limits of detection were 0 and 1800 μg/g. For the assessment of biochemical remission, previously suggested cutoff levels of <250 μg/g [7] for FC and <5 mg/L [21] for CRP were used (FC-based remission and CRP-based remission, respectively). For exploratory analyses, different FC cutoff levels of <50 μg/g [21] and <1000 μg/g were used.

### Sample size

Sample size calculation was based on the power to detect a correlation coefficient that we considered clinically meaningful (*r* ≥ 0.5, resulting in an explained variance ≥25 %). A two-sided Fisher’s *z* test with an alpha of 0.05 will have 90 % power to detect a Pearson’s correlation coefficient of 0.5 when the sample size is 38. Accounting for non-parametric testing (15 %), we aimed to include at least 44 CD patients and 44 UC patients.

### Statistical analysis

Primary analysis was the correlation between levels of FC and clinical indices. Secondary analyses included the correlation between levels of CRP and clinical indices and the correlation of both biomarkers with individual components of disease activity indices. Continuous data with a non-normal distribution were reported using medians and interquartile ranges (IQR). Mann–Whitney *U* tests were used to compare differences between groups, and Spearman’s rank correlations were used to investigate the relation between two parameters with 95 % confidence intervals (CIs) based on bootstrapping with 1000 samples with the same size as the original sample with replacement. The null hypothesis (no correlation between clinical symptoms and biochemical markers of inflammation) was rejected if the 95 % CI of the Spearman’s rank coefficient (*r*_s_) did not include 0. Categorical/dichotomous data were reported as percentages, and Fisher’s exact tests were used to compare differences between groups. Point-biserial correlations were used to investigate the relation between continuous and dichotomous outcomes. Significance was set at *p* < 0.05. Statistical analysis was performed using IBM SPSS Statistics 22 for Windows.

## Results

### Patient characteristics

Between March 2014 and March 2016, a total of 127 patients (49 % male) with a median age of 14.9 years (IQR 13.5–16.4) were included, 82 with a diagnosis of CD and 45 with a diagnosis of UC (Table 1). Based on clinical indices, 79 (63 %) patients were in remission (CD 54 [66 %]; CU 25 [56 %]), 42 (33 %) patients had mild disease activity (CD 27 [33]%; UC 15 [33 %]), and 6 (5 %) patients had moderate disease activity (CD 1 [1 %]; UC 5 [11 %]). No patients had severe disease activity at the time of evaluation.

**Table 1:** Patient characteristics

	CD (*n* = 82)	UC (*n* = 45)
Age (median, IQR)	15.0 (13.1–16.6)	14.2 (11.4–16.7)
Males (*n*, %)	43 (52 %)	19 (42 %)
Months since diagnosis of IBD (median, IQR)	26.1 (9.8–39.1)	22.4 (7.6–43.6)
Current medication for IBD (*n*, %)	77 (93 %)	43 (96 %)
• Anti-TNF (*n*, %)	32 (39 %)	2 (4 %)
• Steroids (*n*, %)	7 (9 %)	9 (20 %)
• Thiopurine (*n*, %)	36 (44 %)	16 (36 %)
• Methotrexate (*n*, %)	8 (10 %)	0 (0 %)
• 5-ASA (*n*, %)	9 (11 %)	37 (82 %)
Previous IBD-related surgery (*n*, %)	12 (15 %)	1 (2 %)
• Perianal surgery (*n*, %)	7 (8 %)	0 (0 %)
• Resectional surgery (*n*, %)	5 (6 %)	1 (2 %)
CD: age at diagnosis (Paris classification)		
• A1a 0–<10 years (*n*, %)	25 (30 %)	
• A1b 10–<17 years (*n*, %)	57 (70 %)	
• A2 17–40 years (*n*, %)	0 (0 %)	
CD: location^a^ (Paris classification)		
• L1 (*n*, %)	7 (9 %)	
• L2 (*n*, %)	24 (29 %)	
• L3 (*n*, %)	49 (60 %)	
• L4a (*n*, %)	34 (41 %)	
• L4b (*n*, %)	3 (4 %)	
• L4ab (*n*, %)	1 (1 %)	
CD: behavior (Paris classification)		
• B1 non-structuring, non-penetrating (*n*, %)	69 (84 %)	
• B2 structuring (*n*, %)	7 (9 %)	
• B3 penetrating (*n*, %)	5 (6 %)	
• B2B3 penetrating and structuring (*n*, %)	1 (1 %)	
• *p* perianal disease (*n*, %)	13 (16 %)	
CD: growth impairment (Paris classification)		
• Evidence of growth delay (*n*, %)	14 (17 %)	
CD: aPCDAI (median, IQR)	5 (0–10)	
UC: extent (Paris classification)		
• E1 proctitis (*n*, %)		5 (11 %)
• E2 left-sided disease (*n*, %)		11 (24 %)
• E3 extensive disease (*n*, %)		6 (13 %)
• E4 pancolitis (*n*, %)		23 (51 %)
UC: severity^b^ (Paris classification)		
• S1 ever severe (*n*, %)		9 (20 %)
UC: PUCAI (median, IQR)		5 (0–15)

*aPCDAI* abbreviated Pediatric Crohn’s Disease Activity Index, *CD* Crohn’s disease, *PUCAI* Pediatric Ulcerative Colitis Activity Index, *UC* ulcerative colitis

^a^L1 distal 1/3 ileum ± limited cecal disease, L2 colonic, L3 ileocolonic, L4a upper disease proximal to ligament of Treitz, L4b upper disease distal to ligament of Treitz and proximal to distal 1/3 ileum

^b^Defined as ever a PUCAI ≥65 points

In total, 48 (38 %) patients were in FC-based remission, of whom 13 (27 %) had clinically active disease. An overview of these patients’ symptoms is provided in the Online Resource.

### Biochemical markers in Crohn’s disease

#### Fecal calprotectin

**Fig. 1:**
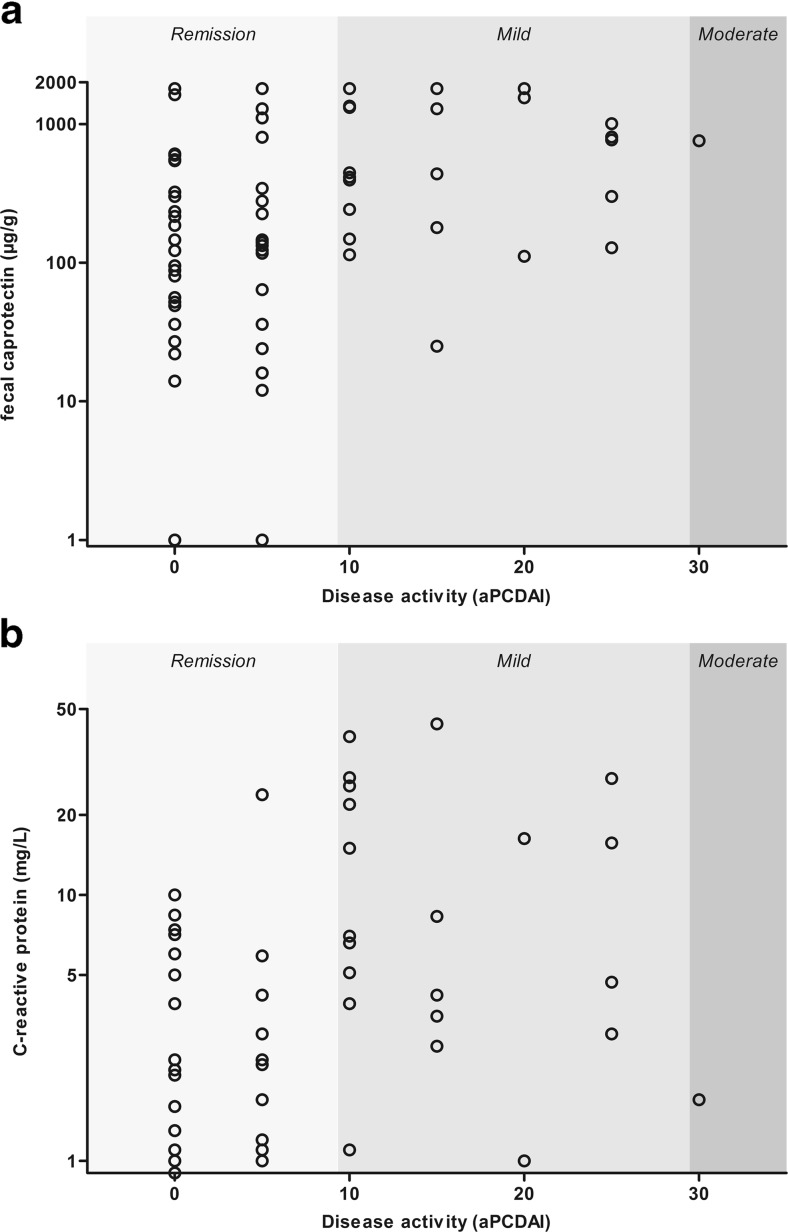
**a** Fecal calprotectin and **b** C-reactive protein levels by clinical disease activity as assessed by aPCDAI

The median FC level in patients with CD was 260 μg/g (IQR 76–1297; range 1–1800 μg/g). The proportion of patients with CD in FC-based remission was 50 %. The proportion of patients with CD in clinical remission with FC-based remission was highly dependent on the cutoff for biochemical remission and was higher compared to patients with clinically active disease (Table 2). Fecal calprotectin had a weak but significant positive correlation with the total aPCDAI score (*r*_s_ = 0.32 [95 % CI 0.13–0.49]; *p* = 0.003; Fig. 1a). Furthermore, FC levels had a weak positive correlation with the aPCDAI components abdominal examination (*r*_s_ = 0.23 [95 % CI 0.02–0.40]; *p* = 0.037) and perirectal disease (*r*_s_ = 0.23 [95 % CI 0.10–0.34]; *p* = 0.036; Table 3).

**Table 2:** The distribution of biochemical disease activity in clinical remission or active disease

CD	Clinical remission^a^ (*n* = 54)	Active disease^b^ (*n* = 28)	*p* value
FC < 50 μg/g	15 (28 %)	1 (4 %)	0.008
FC < 250 μg/g	34 (63 %)	7 (25 %)	0.002
FC < 1000 μg/g	43 (80 %)	15 (54 %)	0.021
CRP < 5 mg/L	45 (83 %)	15 (54 %)	0.008
UC	Clinical remission^a^ (*n* = 25)	Active disease^b^ (*n* = 20)	*p* value
FC < 50 μg/g	4 (16 %)	2 (10 %)	0.678
FC < 250 μg/g	12 (48 %)	6 (30 %)	0.359
FC < 1000 μg/g	23 (92 %)	10 (50 %)	0.002
CRP < 5 mg/L	20 (80 %)	15 (75 %)	0.731

*CD* Crohn’s disease, *CRP* C-reactive protein, *FC* fecal calprotectin, *UC* ulcerative colitis

^a^Abbreviated Pediatric Crohn’s Disease Activity Index <10 or Pediatric Ulcerative Colitis Activity Index <10

^b^Abbreviated Pediatric Crohn’s Disease Activity Index <10 or Pediatric Ulcerative Colitis Activity Index <10

**Table 3:** Correlation between the clinical indices total scores, individual components, and biochemical markers of disease activity

CD (aPCDAI)	FC (*r*_s_)	CRP (*r*_s_)
Abdominal pain^a^	0.133	0.180
Stools (per day)^a^	0.156	0.020
Patient functioning^a^	0.177	0.242*
Weight^a^	0.185	0.052
Abdominal examination^a^	0.231*	0.326*
Perirectal disease^a^	0.232*	0.023
Total aPCDAI score^a^	0.324*	0.275*
UC (PUCAI)	FC (*r*_s_)	CRP (*r*_s_)
Abdominal pain^a^	0.147	−0.039
Rectal bleeding^a^	0.192	0.057
Stool consistency of most stools^a^	0.203	0.121
Number of stool per 24h^a^	0.270	0.140
Nocturnal stools (any episodes causing wakening)^b^	0.239	0.190
Activity level^a^	0.461*	−0.008
Total PUCAI score^a^	0.361*	0.005

*aPCDAI* abbreviated Pediatric Crohn’s Disease Activity Index, *CD* Crohn’s disease, *CRP* C-reactive protein, *FC* fecal calprotectin, *PUCAI* Pediatric Ulcerative Colitis Activity Index, *UC* ulcerative colitis

*Significant correlation (*p* < 0.05)

^a^Spearman’s rank correlations

^b^Point-biserial correlation

#### C-reactive protein

The median CRP level in patients with CD was 1.3 mg/L (IQR 0.4–5.0; range 0.3–44.0 mg/L). The proportion of CD patients in CRP-based remission determined with CRP was 73 %. Crohn’s disease patients in clinical remission were more frequently in CRP-based remission, compared to patients with active disease (Table 2). C-reactive protein had a weak positive correlation with the total aPCDAI score (*r*_s_ = 0.28 [95 % CI 0.05–0.46]; *p* = 0.012; Fig. 1b). Furthermore, CRP levels had a weak positive correlation with the aPCDAI components abdominal examination (*r*_s_ = 0.33 [95 % CI 0.14–0.48]; *p* = 0.003) and patient functioning (*r*_s_ = 0.24 [95 % CI 0.01–0.45; *p* = 0.028; Table 3). In CD patients, CRP levels had a moderate positive correlation with FC levels (*r*_s_ = 0.459 [95 % CI 0.27–0.62]; *p* < 0.001).

### Biochemical markers in ulcerative colitis

#### Fecal calprotectin

**Fig. 2:**
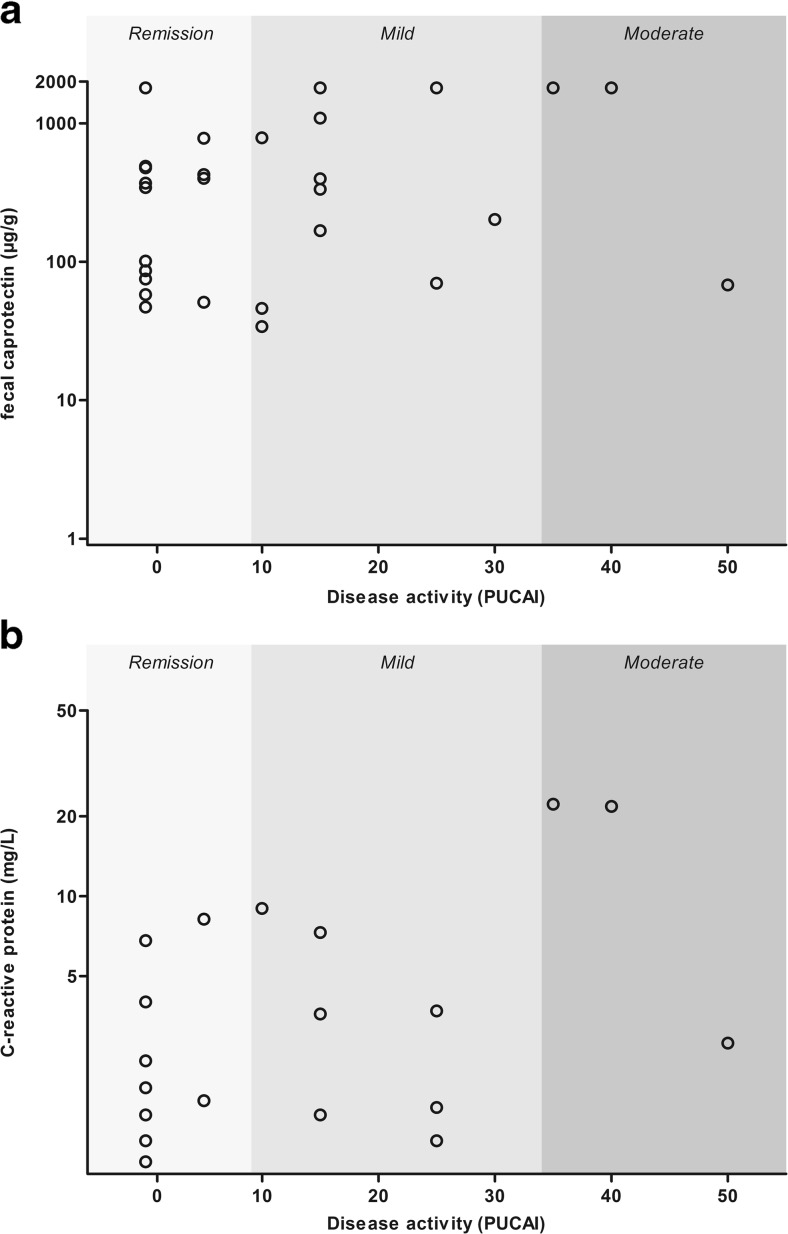
**a** Fecal calprotectin and **b** C-reactive protein levels by clinical disease activity as assessed by PUCAI

The median FC level in patients with UC was 398 μg/g (IQR 73–1290; range 1–1800 μg/g). The proportion of patients with UC in FC-based remission was 40 %. Similar to the analysis in CD, the proportion of patients with UC in clinical remission with FC-based remission was highly dependent on the cutoff for biochemical remission. However, no significant difference was found in biochemical remission rate between patients with and without clinically active disease (Table 2). Fecal calprotectin had a weak positive correlation with the total PUCAI score (*r*_s_ = 0.36 [95 % CI 0.07–0.61]; *p* = 0.015; Fig. 2a and Table 3). Furthermore, the activity level component of the PUCAI had a moderate positive correlation with levels of FC (*r*_s_ = 0.46 [95 % CI 0.20–0.68]; *p* = 0.001; Table 3).

#### C-reactive protein

The median CRP level in patients with UC was 1.5 mg/L (IQR 0.5–4.2; range 0.1–22.2 mg/L). The proportion of UC patients in CRP-based remission was 78 %. There was no difference in the proportion of patients in CRP-based remission, in patients with or without clinically active UC (Table 2). No significant correlation was observed between the total PUCAI score (*r*_s_ 0.01 [95 % CI −0.34–0.30]; *p* = 0.961) or individual PUCAI components and CRP levels (Fig. 2b and Table 3). In UC patients, CRP levels showed no significant correlation with FC levels (*r*_s_ = 0.09 [95 % CI −0.21–0.39]; *p* = 0.542).

## Discussion

In this study, we evaluated the relationship of biomarkers of disease activity with clinical symptoms in children and adolescents who were previously diagnosed with IBD. In both CD and UC patients, the total disease activity index score had a weak positive correlation with FC levels. Only the aPCDAI components abdominal examination and perirectal disease and the PUCAI component activity level correlated significantly with FC levels, albeit weakly. In CD patients, CRP had a weak positive correlation with the total aPCDAI score and the aPCDAI components abdominal examination and patient functioning. In UC patients, CRP did not correlate with total disease activity index score nor with its individual components.

The correlation between FC levels and the total aPCDAI score in this study is lower than the correlation of FC levels with the original Pediatric Crohn’s Disease Activity Index (PCDAI) that is reported in previous studies [1, 9, 10, 15, 13], although results have not been unequivocal [4, 33]. This may be related to the fact that the aPCDAI consists only of clinical components, as opposed to the original PCDAI, which also includes laboratory parameters. The exclusion of these additional parameters from the clinical disease activity score may have reduced the correlation with FC levels, since they are known to correlate with FC levels [7]. In this study, we chose to use the aPCDAI because it is a measure of purely *clinical* disease activity, as opposed to the original PCDAI.

Of the individual aPCDAI components, only abdominal examination and perirectal disease correlated with FC levels. This may be explained by the more objective nature of these observations, compared to the more subjective components such as abdominal pain and patient functioning. However, in studies of adults with CD, no correlation was found between FC levels and perirectal disease [30]. In addition, the more objective component stool consistency of the aPCDAI was not related to levels of FC. Surprisingly, activity level, arguably the most subjective component of the PUCAI, was the only component that correlated significantly with FC level. Similarly, activity level expressed as the level of experienced fatigue was previously found to correlate positively with levels of biochemical disease activity in adult IBD patients [42].

Overall, only a weak correlation was found between clinical disease activity and levels of FC, probably the most sensitive currently available marker for gastrointestinal inflammation in IBD. Even the upper limits of the 95 % CIs of the correlation coefficients (0.51 for CD and 0.62 for UC) indicate that, at most, only a small proportion (respectively, 26 and 37 %) of variation in symptoms can be explained by variation in inflammation. This may partly be explained by the fact that disease activity was relatively mild in our study population. Indeed, the majority of patients were in clinical remission. Consequently, the relatively small variation in the total clinical indices scores potentially decreased the strength of the correlation. However, the distribution of disease activity in our study appears to be similar to that of large cohort studies on the clinical course of pediatric IBD [22, 11]. Thus, the correlation that we found is more likely to reflect the relationship between symptoms and the degree of intestinal inflammation in daily practice, compared to previously reported correlation coefficients from studies that also included patients at the time of diagnosis [9, 1, 4].

The lack of correlation between levels of CRP and clinical disease activity in patients with UC can be explained by the poor sensitivity of CRP for endoscopically active UC [29, 25]. The reason why CRP seems to be less accurate in UC compared to CD is unclear, although it may be explained by the fact that in UC, inflammation is limited to the mucosa, in contrast to the transmural inflammation in CD [38]. Moreover, serum levels of IL-6, the main mediator of the production of CRP, are shown to be higher in children with CD compared to those with UC [5].

In our study, a large proportion of patients had elevated serum and fecal markers of inflammation, despite the complete absence of clinical symptoms. This may be partly explained by the suboptimal accuracy of these biomarkers. However, in our opinion, these elevated inflammatory markers in patients with quiescent IBD probably reflect ongoing IBD activity in the majority of patients. Indeed, many studies have shown that children and adults with IBD in clinical remission have ongoing inflammation on endoscopy [39, 23, 8, 14, 41, 40].

The discrepancy between clinical symptoms and biochemical markers of inflammation may have important implications for the management of IBD. Since symptoms are only weakly associated with the underlying inflammatory process, one could argue whether it should be a prerequisite that a therapeutic agent reduces *both* clinical symptoms and the underlying inflammation. Perhaps these are two distinct aims, potentially with two distinct interventions or treatment strategies. Future research is required to determine the optimal strategy to attain both goals.

The strength of our study is that we included a random sample of children and adolescents with an established diagnosis of IBD. Consequently, our findings are likely to reflect the relationship between disease activity and the degree of intestinal inflammation in daily practice. A weakness is that we did not assess disease activity using endoscopy with histology, since in our practice, endoscopy is not routinely performed to monitor disease activity during follow-up.

In conclusion, in this study of children with previously diagnosed IBD, clinical disease activity was only weakly associated with inflammatory markers FC and CRP. This may implicate that clinical symptoms and inflammatory markers represent two distinct aspects of disease activity and should be considered separately in clinical decision-making.

## Electronic supplementary material

ESM 1 (DOCX 19 kb)

ESM 2 (DOCX 19 kb)

ESM 3 (DOCX 19 kb)

## Abbreviations

aPCDAI: Abbreviated Pediatric Crohn’s Disease Activity Index
CD: Crohn’s disease
CRP: C-reactive protein
FC: Fecal calprotectin
PUCAI: Pediatric Ulcerative Colitis Activity Index
UC: Ulcerative colitis
