# Supplementary material for: Relationship of clinical symptoms with biomarkers of inflammation in pediatric inflammatory bowel disease
Source: Eur J Pediatr. 2016 Aug 29;175(10):1335–42. doi: 10.1007/s00431-016-2762-2 (PMC5031739; doi:10.1007/s00431-016-2762-2)
Supplement: Supplementary file 1 — (DOCX 19 kb) [file 431_2016_2762_MOESM1_ESM.docx]

| *(1) Abdominal pain* | |
| --- | --- |
| No pain | 0 |
| Pain can be ignored | 5 |
| Pain cannot be ignored | 10 |
| *(2) Rectal bleeding* | |
| None | 0 |
| Small amount only, in <50% of stools | 10 |
| Small amount with most stools | 20 |
| Large amount (>50% of the stool content) | 30 |
| *(3) Stool consistency of most stools* | |
| Formed | 0 |
| Partially formed | 5 |
| Completely unformed | 10 |
| *(4) Number of stools per 24 h* | |
| 0–2 | 0 |
| 3–5 | 5 |
| 6–8 | 10 |
| >8 | 15 |
| *(5) Nocturnal stools (any episode causing wakening)* | |
| No | 0 |
| Yes | 10 |
| *(6) Activity level* | |
| No limitation of activity | 0 |
| Occasional limitation of activity | 5 |
| Severe restricted activity | 10 |
| Sum of PUCAI (0–85) |  |

Online Resource table 1. Pediatric Ulcerative Colitis Activity Index (PUCAI)

Turner D, Otley AR, Mack D, Hyams J, de Bruijne J, Uusoue K, Walters TD, Zachos M, Mamula P, Beaton DE, Steinhart AH, Griffiths AM (2007) Development, validation, and evaluation of a pediatric ulcerative colitis activity index: a prospective multicenter study. Gastroenterology 133:423–32. doi: 10.1053/j.gastro.2007.05.029
