# Supplementary material for: Relationship of clinical symptoms with biomarkers of inflammation in pediatric inflammatory bowel disease
Source: Eur J Pediatr. 2016 Aug 29;175(10):1335–42. doi: 10.1007/s00431-016-2762-2 (PMC5031739; doi:10.1007/s00431-016-2762-2)
Supplement: Supplementary file 2 — (DOCX 19 kb) [file 431_2016_2762_MOESM2_ESM.docx]

| *(1) Abdominal pain* | |
| --- | --- |
| None | 0 |
| Mild - Brief, does not interfere with activities | 5 |
| Mod/severe-daily, longer lasting affects activities, nocturnal | 10 |
| *(2) Stools (per day)* | |
| 0-1 liquid stools, no blood | 0 |
| Up to 2 semi-formed with small blood, or 2-5 liquid | 5 |
| Gross bleeding, or ≥6 liquid, or nocturnal diarrhea | 10 |
| *(3) Patient Functioning, General Well-Being* | |
| No limitation of activities, well | 0 |
| Occasional difficulty in maintaining appropriate activities, below par | 5 |
| Frequent limitation of activity, very poor | 10 |
| *(4) Weight* |  |
| Weight gain or voluntary weight stable/loss | 0 |
| Involuntary weight stable, weight loss 1-9% | 5 |
| Weight loss ≥ 10% | 10 |
| *(5) Abdomen* |  |
| No tenderness, no mass | 0 |
| Tenderness, or mass without tenderness | 5 |
| Tenderness, involuntary guarding, definite mass | 10 |
| *(6) Perirectal disease* |  |
| None, asymptomatic tags | 0 |
| 1-2 indolent fistula, scant drainage, no tenderness | 5 |
| Active fistula, drainage, tenderness, or abscess | 10 |
| Sum of aPCDAI (0–60) |  |

Online Resource table 2. Abbreviated Pediatric Crohn’s Disease Activity Index (aPCDAI),

Loonen HJ, Griffiths AM, Merkus MP, Derkx HHF (2003) A critical assessment of items on the Pediatric Crohn’s Disease Activity Index. J Pediatr Gastroenterol Nutr 36:90–5.
