# Supplementary material for: Relationship of clinical symptoms with biomarkers of inflammation in pediatric inflammatory bowel disease
Source: Eur J Pediatr. 2016 Aug 29;175(10):1335–42. doi: 10.1007/s00431-016-2762-2 (PMC5031739; doi:10.1007/s00431-016-2762-2)
Supplement: Supplementary file 3 — (DOCX 19 kb) [file 431_2016_2762_MOESM3_ESM.docx]

|  | Patients with active disease despite FC-based remission |
| --- | --- |
| PUCAI components | Ulcerative colitis (n=6) |
| - Abdominal pain | 3 (50%) |
| - Rectal bleeding | 3 (50%) |
| - Stool consistency | 4 (67%) |
| - Number of stools per 24h | 3 (50%) |
| - Nocturnal stools | 1 (17%) |
| - Activity level | 2 (33%) |
| aPCDAI components | Crohn’s disease (n=7) |
| - Abdominal pain | 2 (29%) |
| - Stools (per day) | 3 (14%) |
| - Patient functioning | 5 (72%) |
| - Weight | 3 (43%) |
| - Abdomen | 1 (14%) |
| - Perirectal disease | 0 (0%) |
| Online Resource table 3. An overview of symptoms contributing to a clinical classification of active disease despite FC-based remission (< 250 µg/g). Data represent the number of subjects with a score on the individual disease activity index components of >0. | |
